# President’s Address before the U. S. V. M. A.1Read before the Thirty-second Annual Meeting of the U. S. Veterinary Medical Association, September 10, 11, and 12, 1895.

**Published:** 1895-11

**Authors:** W. Horace Hoskins


### THE PRESIDENT'S ADDRESS.[1]

BY W. HORACE HOSKINS, D.V.S.

FELLOW-MEMBERS, DELEGATES, AND VISITORS: We open this morning the thirty-second annual meeting of this Association, the parent of all the various State and local Associations in this great and wonderful land. We have for the first time set foot on soil that all will now acknowledge is Western territory, and we this year, for the first time, hold our annual gathering as a Western one. As early as 1884 this Association turned its face Westward, and met in Cincinnati, Ohio, there to receive at the hands of several veterinarians a warm and royal welcome; but as a national meeting the attempt was a dire failure. It was a hard task for the more aggressive and younger element of the Association to win the meeting of 1890 for Chicago, and every possible foreboding was predicted and foretold, all of which came to naught, for it was really one of the most successful meetings we have ever held. Our faces were again turned westward in 1893 at the World's Columbian Congress, where were gathered together during the period of the World's Fair the greatest body of intellectual giants that has ever been drawn together at one spot in the history of the world. Among these great luminaries of intelligence there were none who represented so wide a scope of usefulness, who held greater responsibilities in their keeping, who stand for more in the world's health, wealth, and happiness than the veterinarians who mingled there in their deliberations and efforts to solve some of the most trying problems of the greatest vital importance. Their work was well done, and the fruits of their labors grow more clear and grander day by day. That meeting of 1893, with its wealth of pleasant memories, made possible this meeting to-day at a still more western point, and equally well may it be said that it gave birth to a real national association in the scope of its work as well as in name, and to-day this birth has been sealed for all time to come, for the Pacific Coast, turning its face from the Orient, greets those in the spirit of friendly emulation to do the most

[1] Read before the Thirty-second Annual Meeting of the U. S. Veterinary Medical Association, September 10, 11, and 12, 1895.

good with her *confrères* of the Atlantic Coast, whose faces, turned from the rising sun, now meet on central territory and join thoughts and emotions in one common effort to do the most good for the best people of the grandest country on the earth. This meeting will decide in great measure the place for 1897, when it will be the period for our next western pilgrimage. My earnest wish and longing is that this gathering may make it possible for us to meet on the Pacific Coast, there to have join us in all the breadth and scope of our responsibilities many of our fellow-workers who have already done yeoman service toward a higher and better recognition of the veterinary profession, and won for themselves and their calling a generous and warm recognition of their worth and power.

It is now my pleasure to join with those who have preceded me in conveying to you, one and all, a most sincere and cordial welcome to the sessions of this convention, and to assure you that I feel and fully realize that there is not one within hearing of my voice but who is able to shed some light and some knowledge on the many important subjects that will be ours to consider, and we most earnestly request of you the fullest, freest, and widest discussion of these all-important subjects.

Perhaps it might be better said that no calling in the history of any new country has had a slower growth than ours (though second to none in importance), and equally true that, once out of its swaddling clothes, no other science has had to meet and solve as many trying problems in a few years of its latter-day growth than the profession of veterinary science. The very foundations of our education have been shaken from centre to circumference, and is still one of the most pressing problems for our earnest, thoughtful, and serious consideration.

The field of sanitary science and police, almost wholly within the scope of our work, finds us ill prepared to deal intelligently, scientifically, usefully, and economically with the many pressing questions for solution, to insure to our people the greatest safety and freedom from the dangerous foes that lie therein, and which have too long sapped the vitality, power, progress, and wealth of our people, and destroyed thousands of noble lives that were charged to providential circumstances, and to which there was a common acknowledgment that their time had come, while the death-dealing enemies of life in many directions went relentlessly on gathering in their victims, until the brain of scientific sanitary medicine, hand-in-hand with pathological, microscopical, bacteriological laboratory research, laid bare the causes, and, pointing to the results, suggested the remedies of true medical science and directed the way toward preventative medicine, the true field and goal of all our labors.

The past year has surely been a trying one to those in the routine work of our profession, and following as it did at least two others when the live-stock industry was on the decline, with the generally demoralized business the world over, will make it many months, and in some sections several years, before recovery. Still, it has not been without its blessings, and, hidden deep as they were, we seemed a long while realizing how narrow we had unconsciously allowed the field of our employment to be restricted to. It has taught well the lesson that no science covers so great a field of usefulness and good as our own calling; and while we consider the relief of suffering and the cure of ills in the equine species the acme of all our work, we now, with a less obscured vision, realize that this is only an accessory part of our work. But high and above it all stands the domain of sanitary veterinary science and police, unlimited in its fields, but faintly appreciated in its far-reaching influences; a field over whose border we have just trod, but which now from this day forth must grow in magnitude and in importance until the field of production, the lines of transportation, the centre of its preparation, and the field of sale to the consumer must be policed with a cordon of guards from the ranks of veterinarians, and thus grant to our people the great untold and far-reaching benefits of a wholesome food-supply.

The bacteriological laboratory points the way and beckons hosts to do its work that the hidden mysteries of disease, infection, contagion, modes of transmission, etc., must be solved, that our people may be preserved from the ravages caused and enhanced by micro-organisms of a dangerous and insidious character.

Already an insufficient number of such men exist in our profession, and our schools ten years ago feebly equipped their graduates for such positions as these. Only now are our institutions of learning grasping a full conception of the situation and grading and broadening their curricula to equip their students properly.

Two years ago I saw fit, from this Association, as one of its officers, to criticise strongly the Department of Agriculture at

Washington for yielding to the pernicious influences of politicians, displacing from positions of great importance good and well-tried men and filling their places with untrained and inexperienced members of the profession unfamiliar with the nature of the duties and skill required. With as much earnestness and with great pleasure and satisfaction I to-day congratulate Secretary Morton and honor him above all other men of the present administration for the establishment in his department of a true civil-service, and the spirit of fairness and courage he has shown in this direction will be remembered by those who are zealous for their country's good long after he has laid down the cares of office to his successor. And let it be remembered that this merit-system of appointments is the only concession we have asked as an Association, believing that by this means and this channel only could we give to our government and the people it represents a true and just idea of the importance of our calling to the nation, the value of our services in the preservation of animal life, and the benefits to a race of people in health, wealth, and prosperity. And one word more: let it be understood that the establishment of this true civil-service and merit system of appointment will never be removed. No successor in office will dare to ignore its establishment. They may not believe in the spirit of this reform; they may sneer at the policy of it and may strain its provisions to hinder its growth and perpetuation, but woe be unto the one who dares to place himself above this law! It has come to stay because it is right, because it is fair, because it is just; and let me ask of every member of this Association, whose zeal and interest I am sure are not second to my own, that no hand or voice from one of our number be ever raised or spoken to stay the progress of this movement that promises so much for the profession's advancement in every true and proper light.

